# Promoting cessation and a tobacco free future: willingness of pharmacy students at the University of Lagos, Nigeria

**DOI:** 10.1186/1617-9625-5-13

**Published:** 2009-08-22

**Authors:** Bolajoko A Aina, Adebayo T Onajole, Babatunde MO Lawal, Opeoluwa O Oyerinde

**Affiliations:** 1 Department of Clinical Pharmacy and Biopharmacy, Faculty of Pharmacy, University of Lagos, Idi Araba Campus, Lagos, Nigeria; 2 Department of Community Health, College of Medicine, University of Lagos, Idi Araba Campus, Lagos, Nigeria; 3 Medical Department, UACN Plc, UAC House, 1-5 Odunlami St., Lagos, Nigeria; 4 Pharmacy Department, Wesley Guild Hospital, Ilesha, Oshun State, Nigeria

## Abstract

**Background:**

Tobacco use is projected to cause nearly 450 million deaths worldwide during the next 50 years. Health professionals can have a critical role in reducing tobacco use. Therefore, one of the strategies to reduce the number of smoking-related deaths is to encourage the involvement of health professionals in tobacco-use prevention and cessation counseling. As future health care providers, pharmacy students should consider providing assistance to others to overcome tobacco use and be involved in promoting a tobacco free future as part of their professional responsibility.

This research was to determine the knowledge of tobacco/smoking policy, willingness to be involved in tobacco cessation, attitude to keeping a tobacco free environment and the smoking habit among pharmacy students at the University of Lagos.

**Methods:**

Data was collected by the use of self administered questionnaire which was aimed at assessing their smoking habit, determining their knowledge and attitude to smoking policy and willingness to be involved in smoking cessation. The population sample was all the pharmacy students in their professional years (200 to 500 Levels) at Idi-Araba Campus of the University of Lagos.

**Results:**

Out of 327 qualified participants, 297 responded to the questionnaire which was about 91% participation rate but out of these only 291 questionnaires were useful which came to 89%.

There seemed to be no statistically significant difference between the smoking habits among the different levels (p > 0.05). Overall, the current smoking prevalence was 5.5% which is lower than the national prevalence rate of 8.9%.

Awareness of WHO FCTC global tobacco treaty was low (9.3%) among pharmacy students but they agreed that pharmacists and pharmacy students should be involved in quit smoking program (93.1%) and they were willing to be involved in helping smokers to quit (85.9%). Majority agreed that smoking should not be permitted in pharmacies (87.9%) and at pharmacy students' events (86.9%).

**Conclusion:**

From this study it can be concluded that smoking prevalence is low among pharmacy students at the University of Lagos. Awareness of global policy is low but they are willing to be involved in smoking cessation and promoting a tobacco free future.

## Background

Tobacco use is the single largest cause of preventable death in the world today. The *WHO report on the global tobacco epidemic, 2008 *[1] provides a comprehensive analysis, based on data from 135 countries, of patterns of tobacco use, the deaths that result and the measures to reduce deaths.

Tobacco kills a third to a half of all those who use it. On average, every user of tobacco loses 15 years of life. Total tobacco-attributable deaths from ischaemic heart disease, cerebrovascular disease (stroke), chronic obstructive pulmonary disease and other diseases are projected to rise from 5.4 million in 2004 to 8.3 million in 2030, almost 10% of all deaths worldwide. More than 80% of these deaths will occur in developing countries [1].

Tobacco use is highly prevalent in many countries. According to estimates for 2005, 22% of adults worldwide currently smoke tobacco. Some 36% of men smoke compared to 8% of women [2].

WHO recommends five policies for controlling tobacco use: smoke-free environments; support programmes for tobacco users who wish to stop; health warnings on tobacco packs; bans on the advertising, promotion and sponsorship of tobacco; and higher taxation of tobacco. About half of all countries in the world implement none of these five recommended policies, despite the fact that tobacco control measures are cost-effective and proven. Moreover, not more than 5% of the world's population is fully covered by any one of these measures [2].

The World Health Organization has estimated that tobacco and its products kill over 3.5 million people worldwide every year and it is extrapolated that by the decade 2020–2030, tobacco will kill 10 million people a year [3].

The World Health Organization Framework Convention on Tobacco Control (WHO FCTC) is a global public health treaty aimed at reducing the burden of disease and death caused by tobacco consumption. The WHO Framework Convention for Tobacco Control (WHO-FCTC), adopted by the 56th World Health Assembly in May 2003, is the first international public health treaty on tobacco control [4]. The Convention opened for signature on 16th June 2003 in Geneva, Switzerland. The Convention quickly became one of the most widely embraced treaties in United Nations history; within two and a half years, it boasted more than 100 Contracting Parties. It officially entered into force in February 2005 and by the end of 2006, the total number of Parties had reached 149 covering more than three quarters of the world's population. At present the Signatories to the WHO FCTC is 168 and the Parties to the WHO FCTC is 166 [5]. It gives the international community tools to stand up to tobacco giants, decrease global addiction rates, and reverse the tobacco epidemic.

The global tobacco treaty bans tobacco advertising, promotion and sponsorship, and insulates public health policy from interference by tobacco corporations. The treaty's advertising ban means an end to some of the tobacco industry's most effective and deadly tactics, like Philip Morris/Altria's Marlboro Man, in countries that ratify. While Philip Morris/Altria, British American Tobacco (BAT) and Japan Tobacco International (JTI) continue to aggressively target developing countries to expand markets for their products, the tobacco giants are renewing their efforts to derail the treaty process in countries around the world.

The global tobacco treaty is a major victory for the corporate accountability movement. It sets important precedents for regulating other abusive industries that profit at the expense of people's health and the environment.

Tobacco use among health professionals is of particular interest in the area of tobacco related surveillance since they are not only responsible for primary health care and education for tobacco related issues such as cessation and exposure to second-hand tobacco smoke, but are also role models in the community. Furthermore, the preamble of the WHO FCTC emphasizes the role of health professional bodies in efforts to include tobacco control in the public health agenda and contribute actively to the reduction of tobacco consumption. These activities are also described in the Code of Practice for Health Professionals which has been officially adopted now by several Health Professional Associations worldwide [6].

Since 1987, the World Health Organization (WHO) has sponsored World No Tobacco Day to encourage countries to implement comprehensive programs to reduce tobacco use. Involvement of health professionals in tobacco control is very importatnt such that the theme of World No Tobacco Day 2005 was 'Health professionals against tobacco'[7].

Pharmacists are healthcare providers involved in treating and preventing illness and promoting health, and are therefore central to achieving the tobacco cessation goals.

The Global Network of Pharmacists Against Tobacco, established by the International Pharmaceutical Federation (FIP) in collaboration with the WHO EuroPharm Forum, is a global forum for pharmacists, pharmaceutical students and their professional organizations as well as other individuals or organizations interested in smoking cessation and tobacco control activities. The network was officially launched during the World Conference on Tobacco or Health 2003 in Helsinki, Finland, in connection with the Pharmacists Special Session [8].

Since the launch of the Global Network of Pharmacists Against Tobacco in Helsinki in August 2003, FIP has been involved in many new Tobacco Cessation initiatives.

During the FIP Congress 2003 in Sydney, FIP adopted a Statement of Policy on the Role of the Pharmacist in Promoting a Tobacco Free Future. The statement includes recommendations both for pharmaceutical organizations and for individual pharmacists to help people who wish to give up smoking or other uses of tobacco, and to encourage others to do so.

As a step towards the implementation of the Statement, the FIP Council agreed to make a combined effort of all FIP Member Organisations to mobilise pharmacists around Tobacco Cessation. This issue was tackled through a global campaign for pharmacists, launched on the World No Tobacco Day on 31 May 2004.

As an offshoot of Code of Practice on Tobacco Control for Health Professional Organisations a meeting of the FIP Global Network of Pharmacists Against Tobacco was held during the 64th FIP Congress in New Orleans, LA, USA, which was attended by more than 70 pharmacists from 20 countries after which FIP issued a Press Release entitled "FIP Calls for Ban on Tobacco Sales and Smoking in Pharmacies".

A Joint Statement was made by the International Pharmaceutical Students' Federation (IPSF) and the European Pharmaceutical Students' Association (EPSA) This joint statement is based on the *WHO Mandate in Public Health *and on the *FIP Statement of Policy on the Role of the Pharmacist in Promoting a Tobacco Free Future*. As future health care providers, pharmacy students should take a proactive role in developing initiatives designed to target the increasing burden of tobacco use.

IPSF and EPSA recognise that pharmacy students, as role models and future members of the health care team, have a crucial role in promoting cessation of tobacco use. Collaboration with other heath care students and professional associations will help ensure a tobacco free future.

Pharmacy students and pharmacists should consider providing assistance to others to overcome this condition as part of their professional responsibility.

Pharmacists have been involved in tobacco cessation activities in some 14 countries [9-11].

Since students in healthcare profession are future professionals, basic information about tobacco smoking among healthcare professional student population would be important since their approach and credibility as future treatment providers may be influenced by their own smoking habits [12]. In a study carried out among health profession students 86.6%–99.8% believed health professionals should advise patients about smoking cessation however only 5.2%–36.6% among pharmacy students had received formal training in tobacco cessation counseling. Among these students 71.7% – 99.0% believed that health professionals should be trained in cessation techniques [13].

Objectives of the study were to assess the prevalence of cigarette smoking among pharmacy students, assess the awareness and attitude to tobacco/smoking control policy and determine the willingness of pharmacy students at the University of Lagos to be involved in tobacco cessation and keeping a tobacco free environment.

## Methods

### Study Area

Study Area was Faculty of Pharmacy of the University of Lagos, Nigeria.

### Study Population

Study population was pharmacy students in their professional years (200 Level to 500 Level). The total student population was 327 but at the end of the study only 297 students participated and 291 questionnaires were useful.

### Study Design

A cross-sectional survey targeting the entire population size

### Study instrument

A well structured, pretested self administered questionnaire was used. The questionnaire of this study was developed based on the questionnaire of Global Health Professional Survey (GHPS) [14].

The questionnaire consisted of demographic characteristics (age, sex, level, and religion.), smoking behaviour and attitudes (smoking status/prevalence, stage and age at which smoking started, number of cigarettes smoked daily), knowledge and attitude to smoking/tobacco policy and curriculum/training,

### Procedure for data collection

Consent of the students was sought before distribution of questionaire. Questionnaires were distributed to the students during lecture time. The questionnaires were filled and retrieved same time.

### Data Analysis

The information in the questionnaires was transferred into Statistical Package for Social Sciences (SPSS 10) software. Data analysis consisted of frequency analysis and cross tabulation for Chi square (X^2^) test for statistical significance.

## Results

In this study out of 327 qualified participants, 297 responded to the questionnaire which was about 91% participation rate but out of these only 291 questionnaires were useful which came to 89%.

Overall smoking prevalence was 5.5% and all these were males.

**Figure 1:**
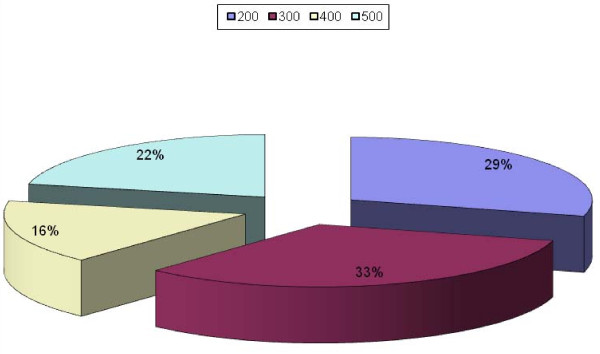
**Percentage each level is contributing to total Respondents**.

Figure 1 shows the percentage each level is contributing to total respondents.

**Figure 2:**
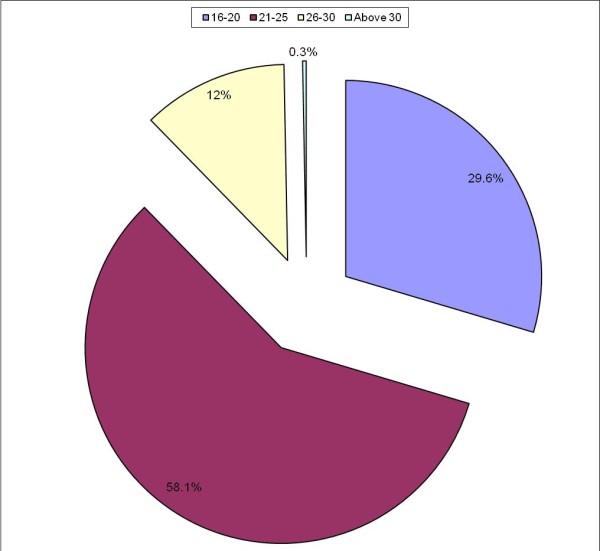
**Age of respondents**.

Majority of the respondents fell within the age bracket of 21–25 (58.1%) followed by age bracket 16–20 (29.6%). Only one respondent was above 30 years of age representing a marginal 0.3% (Figure 2). There was statistically significant difference in the age distribution between the different levels of study (p < 0.05).

**Figure 3:**
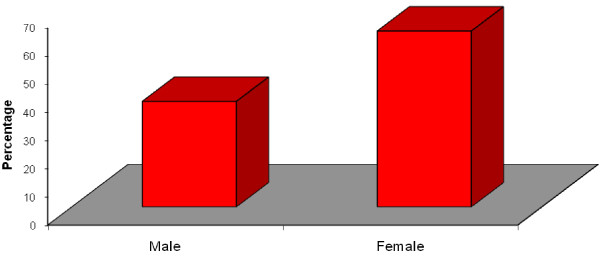
**Sex of respondents**.

Among the respondents 62.5% were females while 37.5% were males (Figure 3).

There was statistically significant difference in the sex distribution between the levels of study (p < 0.05).

**Figure 4:**
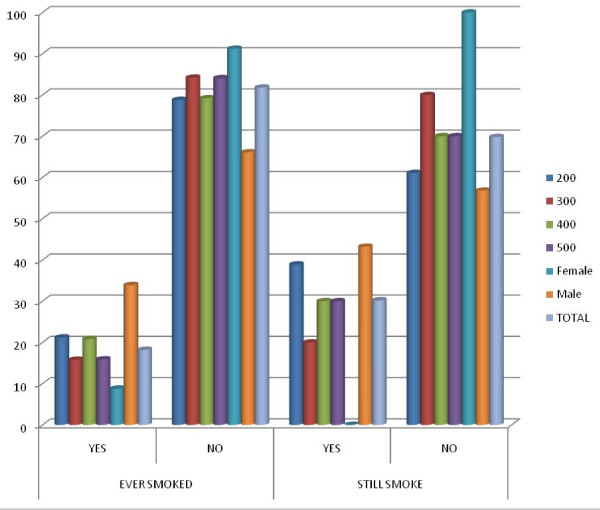
**Respondents that had ever smoked and those that are still smoking among them by level of study and sex**.

Figure 4 shows the smoking status of those that had ever smoked and those still smoking among them according to level of study and sex. There was no statistically significant difference in the smoking status of the different levels (p > 0.05).

Among female respondents 8.8% of them had ever smoked but none of these is still smoking while among the male respondents 33.9% of them had ever smoked and 43.2% of these are still smoking. There was statistically significant difference in the smoking status between male and female respondents (p < 0.05). All the respondents still smoking are males.

**Figure 5:**
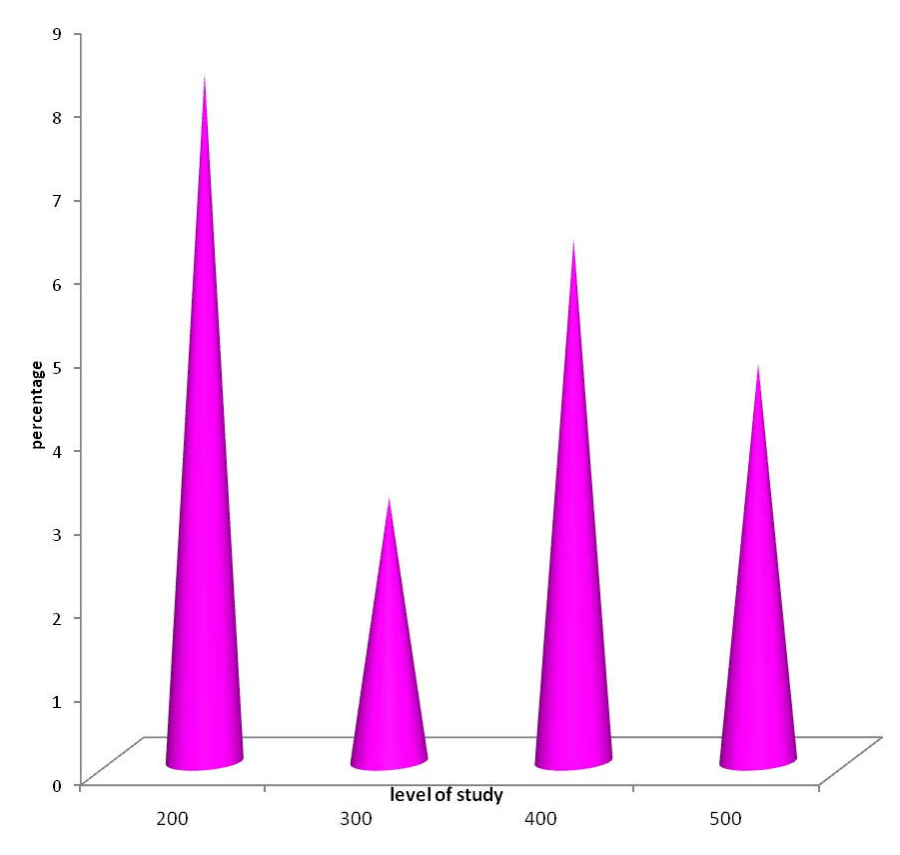
**Current smoking status by level of study**.

The current smoking status was highest in 200 level (8.24%) and lowest in 300 level (3.16%) (Figure 5).

**Figure 6:**
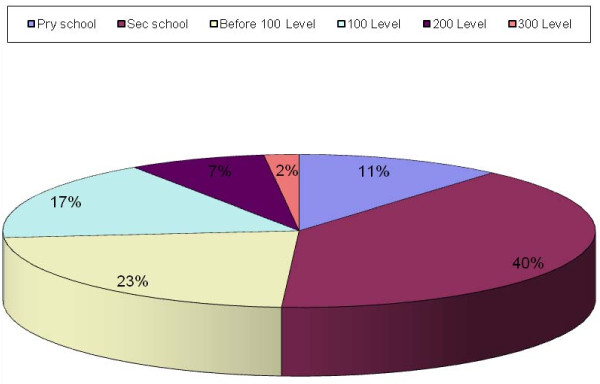
**Stage of starting smoking**.

Majority started smoking at secondary school (39%) and the lowest was in 300 level (Figure 6). There was no statistically significant difference in the stage of starting smoking among the different levels (p > 0.05).

**Figure 7:**
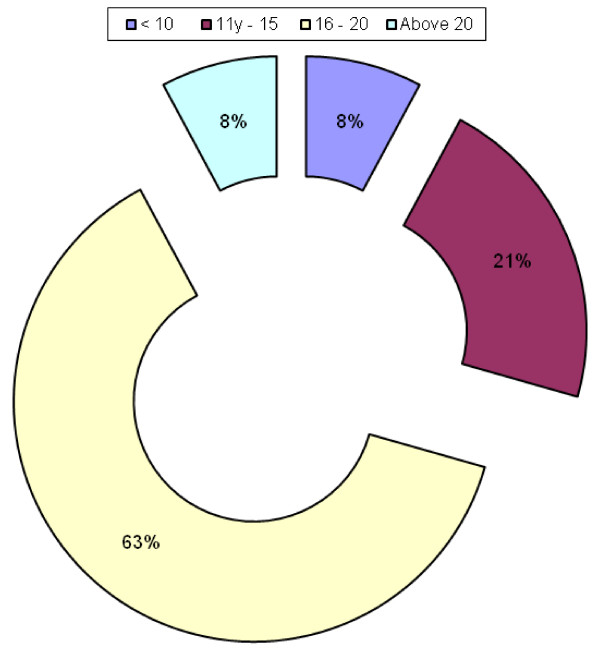
**Age of starting smoking**.

Majority started smoking between ages 16–20 years (62%) and the least were below 10 and above 20 years (8%) (Figure 7). There was no statistically significant difference in the age of starting smoking between the different levels of study (p > 0.05). At each level majority started smoking between ages 16 – 20 years.

Majority were smoking between 1 – 5 cigarettes per day, both among those that have stopped smoking (88.9%) and those that are still smoking (93.7%).

Table 1 shows the positive responses to the awareness questions. Overall 95.9% of them were aware of tobacco induced diseases and at each level of study over 90% of them were aware of tobacco induced diseases. There was no statistically significant difference in the awareness of tobacco induced diseases between the different levels (p > 0.05). Only about 9.0% were aware of World No Tobacco Day. There was no statistically significant difference in the awareness of world no tobacco day among the different levels of study (p > 0.05). Among the respondents about 6.3% were aware of Nigerian Tobacco Decree. There was no statistically significant difference in the awareness of Nigerian tobacco decree among the respondents in the different levels of study (p > 0.05). Only about 9.3% were aware of WHO FCTC. There was no statistically significant difference in the awareness of WHO FCTC among the respondents in the different levels of study (p > 0.05)

**Table 1:** Awareness responses according to level of study

	**200 Level** **n (%)**	**300 Level** **n (%)**	**400 Level** **n (%)**	**500 Level** **n (%)**	**Total** **n (%)**
Aware of Tobacco Induced Diseases	80 (94.1)	92 (91.4)	46 (95.8)	61 (96.8)	279 (95.9)

Aware of World No Tobacco Day	9 (10.6)	9 (9.5)	3 (6.3)	5 (8.2)	26 (9.0)

Aware of Nigeria Tobacco decree	6 (7.1)	8 (8.4)	2 (4.2)	2 (3.7)	18 (6.3)

Aware of WHO FCTC	6 (7.1)	13 (13.7)	3 (6.4)	5 (8.1)	27 (9.3)

Table 2 shows agreement responses to willingness and attitudinal questions.

**Table 2:** Agreement responses to willingness or attitudinal questions according to level of study

**Questions**	**200 Level** **n (%)**	**300 Level** **n (%)**	**400 Level** **n (%)**	**500 Level** **n (%)**	**Total** **n (%)**
Tobacco should not be sold in pharmacies	64 (75.3)	63 (67.0)	40 (83.3)	58 (93.6)	230 (77.9)

No smoking in pharmacy	72 (84.7)	79 (83.2)	44 (91.7)	61 (96.8)	256 (87.9)

pharmacists should not smoke	72 (84.7)	80 (84.2)	38 (79.2)	52 (82.54)	242 (83.2)

Smoking cessation counseling should be in pharmacy curriculum	77 (90.6)	80 (84.2)	40 (85.1)	57 (91.94)	254 (87.9)

No smoking at pharmacy students' events	72 (84.7)	83 (88.3)	40 (83.4)	57 (90.4)	252 (86.9)

It is your duty to be involved in smoking cessation program	76 (89.5)	87 (91.6)	47 (97.9)	62 (98.4)	272 (93.5)

Pharmacists and pharmacy students should be involved in quit smoking program	78 (91.7)	86 (88.5)	46 (95.8)	61 (96.8)	271 (93.1)

Willing to help smokers to quit	71 (83.5)	83 (87.4)	40 (83.3)	55 (88.7)	249 (85.9)

About 94% agreed to the statement that it is their duty as future health care providers to be involved in smoking cessation programme while only 1.4% disagreed. At least 90% of the respondents agreed to this statement at each level of study. There was no statistically significant difference in the level of agreement to this statement between the levels of study (p > 0.05).

There was over 80% agreement to the willingness/attitudinal questions except in the response to tobacco should not be sold in pharmacies where the response was less than 80%.

Also in all the agreement responses to willingness/attitudinal questions there was no statistically significant difference among the levels of study except in the response to tobacco should not be sold in pharmacies.

## Discussion

The major objectives of this survey were to investigate the prevalence of smoking habit among the entire pharmacy students and to determine their awareness and attitude to smoking policy and willingness to be involved in smoking cessation. The overall current smoking prevalence was 5.5% among the respondents and all these were males. This value is better than the report of studies carried out among youths in Cross Rivers state of Nigeria where the prevalence was 23.9% in males, 17.0% in females and 18.1% overall since the pharmacy students fall into the age bracket of youth[15].

The students age group falling among youth is very important to note such that they should be able to influence their peers and the age group is so important that the World no tobacco day for last year (2008) was 'Tobacco-free youth' and the hand bill had the caption 'The tobacco Industries catches you young'; 'Break The Tobacco Marketing Net' and 'Ban all tobacco advertising, promotion and sponsoring for a tobacco free youth' [7].

This result is also comparable to the 3% prevalence Faseru et al obtained in a survey on second to sixth year medical students of University of Ibadan [16].

The prevalence is desirably low especially when compared to what is obtained in similar works done in developed countries, for instance, in a survey done among medical students in a south German University, a prevalence of 17.6% was obtained among female students and 29.2% among male participants [17]. Also in surveys carried out in ten WHO member countries it was found that smoking prevalence was higher than 20% in seven of the 10 countries surveyed [5].

Comparing the smoking prevalence obtained in this study to that of the general Nigerian population which according to 1998 survey [18] was 15% among males and 2% among females one may say smoking is not common among pharmacy students. However, according to Faseru et al [16], there is need to intensify prevention and control efforts because of the potentials of escalation of smoking rates due to aggressive marketing strategies of emerging big tobacco industry.

Also of note is the fact that, these are future healthcare professionals and one smoking pharmacist for instance would have a profound influence on the smoking habits of his hundreds of patients/clients in the general populace. Health professional who continues to smoke sends inconsistent message to patients whom he/she counsels.

Current smoking among the female participants in this survey was nil compared to 5.5% obtained among the male students, this further establishes the fact that tobacco smoking prevalence in Nigeria is lesser among females than males and this means more attention needs to be given to the males in prevention and controlling tobacco smoking.

As regards the distribution of smoking status across course of study, the prevalence is highest among the 200 level students but it is expected that this prevalence would have reduced by the time these ones are getting to higher levels as is shown in Figure 4 whereby the frequency of those still smoking was reduced more in the higher levels.

Majority of those that have actually ever smoked started before entering the University and most especially at the secondary school level, some even started smoking before the age of 10 years this implies that there is need to start health education on harmful effect of smoking at least right from primary school if not kindergarten.

Over 90% of the current smokers smoke between 1–5 cigarettes per day and only 6% smoke over 20 cigarettes per day. About 89% of those that have stopped smoking were smoking between 1–5 cigarettes per day and 5% were smoking 6–10 and 11–15 sticks of cigarette per day. This is important in the sense that it is one of the indicators of nicotine addiction in Fagerstrom nicotine addiction test [19]. From this result it can be said that only about 6% of current smokers might be maximally addicted to nicotine based on Fagerstrom nicotine addiction test.

It should be noted that the less addicted one is, the easier it becomes to quit smoking, so, there is hope that the few current smokers among pharmacy students would be easy to counsel and convince to stop smoking and we might end up having none of the pharmacy students being smokers.

From the result, the response to awareness of Nigeria tobacco decree and WHO FCTC was quite low. The students not being aware of Nigeria tobacco Decree may not be able to counsel and help spread or enforce the decree for example the decree says no smoking in public places [20].

The response to each attitudinal question addressing the main thrust of these local and global policies were quite favourable which implies that there would be minimal effort necessary to convince pharmacy students of the University of Lagos to abide with the code of conduct for health care professionals and also to be in line with year 2007 theme for world no tobacco day which was 'smoke free environments' since majority of the students agreed that there should be no smoking at pharmacy students' events and in pharmacies.

They will serve as good counsellors that the public may rely on or believe in since majority of them do not smoke and also most of them are willing to be involved in smoking cessation program.

Over 85% of pharmacy students agreed that patient counselling about smoking cessation should be included in pharmacy curriculum, this compared favourably with the results of the survey of ten WHO member countries where about 93% thought health professionals should receive cessation counselling training as part of their normal curriculum [13].

Over 93% of pharmacy students agreed that pharmacists and pharmacy students should be involved in quit smoking programme and this is similar to the report of the survey of 10 WHO member countries where about 86 to 99% believed that health professionals should give advice or information about smoking cessation to patients [13]. The result is also similar to the report of survey of pharmacists in Lagos State whereby 98% agreed that pharmacists should be involved in the global campaign against smoking programme [21].

In the results of this study there was no significant difference in the agreement of the students at different levels of study which implied that the students at different levels agreed to the points that patient counselling about smoking cessation should be included in pharmacy curriculum, pharmacists and pharmacy students should be involved in quit smoking programme and are willing to be involved in smoking cessation program.

## Conclusion

From this study it can be concluded that smoking prevalence is low among pharmacy students and is even nil in females at the University of Lagos. The prevalence is highest among the 200 level students

It can also be concluded from the result of this study that though level of awareness of pharmacy students at the University of Lagos of global smoking policy is low but their attitude towards the contents of the policy is highly favourable. They are willing to keep a smoke free environment, promote a tobacco free future and be involved in smoking cessation programme and this cuts across all the levels.

It is being recommended that smoking cessation programme should be included in pharmacy curriculum.

## Competing interests

The authors declare that they have no competing interests.

## Authors' contributions

BAA conceived and designed the study, participated in acquisition, analysis and interpretation of data and was involved with drafting and revising final approval of manuscript. ATO contributed to the design of the study, interpretation of data and was involved in revising the manuscript critically for intellectual content. BMOL contributed to the design of the study, the interpretation of data and was involved in revising the manuscript critically for intellectual content. OOO participated in the acquisition and analysis of data. All the authors gave final approval of the version to be published.
